# Association between Phytochemical Index and Osteoporosis in Women: A Prospective Cohort Study in Korea

**DOI:** 10.3390/nu15071605

**Published:** 2023-03-25

**Authors:** Hyeonji Yoo, Kyong Park

**Affiliations:** Department of Food & Nutrition, Yeungnam University, 280 Daehak-ro, Gyeongsan 38541, Republic of Korea

**Keywords:** women, phytochemicals, osteoporosis, Korean, cohort study

## Abstract

Osteoporosis is a prevalent issue among postmenopausal women, who have a higher incidence of the condition than men. This study aimed to examine the relationship between phytochemical-rich food intake and osteoporosis incidence in premenopausal and postmenopausal women. The data analyzed included 4600 women aged 40–69 who were free of osteoporosis at baseline, with dietary intake evaluated through a semi-quantitative food frequency questionnaire and osteoporosis prevalence determined using interviewer-administered questionnaires and bone mineral density tests. The phytochemical index (PI) was calculated to reflect the intake levels of phytochemical-rich foods. Postmenopausal women in the highest PI quartile had a 16% lower risk of osteoporosis (95% confidence interval (CI): 0.71 to 0.99, *p* for trend = 0.02) than those in the lowest quartile, while no significant association was observed among premenopausal women (hazard ratio: 0.98, 95% CI: 0.78 to 1.24, *p* for trend = 0.8). These findings suggest that consuming phytochemical-rich foods may have a protective effect against osteoporosis in postmenopausal women, offering valuable scientific insights. However, additional research is needed to validate these findings using biochemical data. Overall, this study highlights the potential of dietary interventions to reduce the risk of osteoporosis in postmenopausal women.

## 1. Introduction

As the global population ages, there is a growing concern about the impact of aging-related diseases, particularly osteoporosis [1]. The condition is characterized by reduced bone mass and strength, leading to an increased risk of fractures, disability, and mortality, especially in older adults and postmenopausal women [2]. It is estimated that over 200 million women worldwide have osteoporosis [3,4]. In Korea, the number of patients diagnosed with osteoporosis has surpassed 1 million since 2019, with a majority of these patients being female (94.3%) [5,6]. The cost of caring for individuals with osteoporosis has shown a steady increase and was determined to account for 16.7% of the national healthcare expenditure in Korea, according to a study conducted by the Korea National Health Insurance Agency from 2008 to 2011 [7]. In the US, the projected number of fractures and costs related to osteoporosis are estimated to rise by 50% from 2006 to 2025, with a total cost of $25.3 billion [8]. Osteoporotic fractures can result in complications such as back pain, breathing difficulties, and blood clots, and may also increase the likelihood of death [9]. Therefore, it is crucial to identify dietary factors that can mitigate the impact of aging-related diseases such as osteoporosis.

Although fruit and vegetable intake are commonly associated with positive health outcomes, research findings on their effect on bone health are conflicting [10,11,12]. However, individual phytochemicals, which are plant-based chemical compounds abundant in fruits and vegetables, such as isoflavones, polyphenols, genistein, lycopene, and carotenoids, have been shown to enhance bone mineral density (BMD) and osteogenesis [13,14,15,16,17,18,19,20]. These phytochemicals have various mechanisms of action that contribute to their beneficial effects, including inhibiting osteoclast formation and reducing cytokine damage, promoting bone cell growth, and reducing bone resorption markers. Despite growing evidence indicating the potential health benefits of phytochemicals, community-based epidemiological studies on the overall effects of phytochemical intake and their association with bone health remain limited. This lack of information may be due to the scarcity of food composition databases, which provide crucial details about these metabolites that are needed to test hypotheses about the health benefits of specific plant metabolites in human populations.

The phytochemical index (PI) has been recently proposed as a surrogate marker to reflect the levels of phytochemical intake [21,22]. Several epidemiological studies have shown that higher PI is associated with a reduction in inflammation, hypertension, diabetes, metabolic syndrome, and stroke [21,23,24,25,26]. A recent meta-analysis confirmed that PI is inversely associated with obesity and overweight, with higher PI levels being observed in groups with lower rates of obesity and overweight [27]. Given the promising findings associated with PI, it is possible to use this index to examine the overall effects of phytochemical intake on bone health. By using PI as a marker, it would be feasible to investigate the association between phytochemical intake and bone health in community-based studies and determine the potential role of phytochemicals in preventing or managing osteoporosis.

Osteoporosis is a significant health concern, and the lack of prevention strategies and dietary guidelines is a major issue. Thus, this research aims to address this gap by investigating the longitudinal association between PI and osteoporosis risk in premenopausal and post-menopausal Korean women, using the Korean Genome and Epidemiology Study (KoGES). The findings of this study will contribute to the understanding of the potential benefits of phytochemicals in preventing or managing osteoporosis, which could inform the development of dietary guidelines for osteoporosis prevention in this population.

## 2. Materials and Methods

### 2.1. Study Population

This study was performed using data from the KoGES Korea Association Resource (KARE) study. The KARE study is a cohort of individuals aged 40–69 residing in Ansan and Anseong, Gyeong-gi-do, who were followed every two years since the baseline survey conducted between 2001 and 2002 [28]. Further details of the KARE study can be found in previous literature [29]. The KARE study recruited a total of 10,030 participants: 5012 from Ansan and 5018 from Anseong. This analysis covered the period from the baseline survey to the fifth follow-up, excluding the following: (1) men (*n* = 4758); (2) those with a daily energy intake less than 500 kcal or more than 5000 kcal (*n* = 202); (3) those diagnosed with osteoporosis at baseline who had a history of osteoporosis, treatment received, and medication use (*n* = 169); and (4) those diagnosed with diabetes at baseline (*n* = 301). The final analysis included 4600 women, with 2755 in pre-menopause and 1845 in menopause. The data collection and analysis for this study were approved by the Institutional Review Board (IRB) of the Korea Disease Control and Prevention Agency (IRB number: KU-IRB-15-EX-256-A-1) and the Ethics Committee of Yeungnam University (IRB number: 7002016-E-2016-003).

### 2.2. General Characteristics and Health Information of the Participants

The study collected information on participants’ demographics and lifestyle habits through survey questionnaires administered by trained professionals [30]. The survey included questions on average monthly family income, which was categorized as <0.5, 1–2, 2–4, and ≥4 million won, and it was reclassified into low or mid–low, and mid–high or high for statistical analysis.

Education level was also recorded and classified into high school graduates or lower, and college graduate or higher. The physical activity level of participants was determined using a questionnaire that assessed the types, duration, and frequency of physical activity performed. Metabolic equivalents of tasks (METs-hours/week) were calculated by assigning a weight based on exercise intensity to the time spent exercising/day, enabling the determination of physical activity levels [31], and were divided into tertiles (low, mid, and high). Alcohol consumption status was classified as either non-drinking or drinking, while smoking status was divided into current smoking, former smoking, and non-smoking for the analysis.

Anthropometric measurements, including height and weight, were taken with participants standing naturally with their eyes facing forward and without shoes on. Height was recorded in centimeters and weight in kilograms [30]. The body mass index (BMI) was calculated by dividing the participant’s weight in kilograms by the square of their height in meters.

### 2.3. Dietary Assessment

The participant’s dietary intake was assessed using the semi-quantitative food frequency questionnaire (SQFFQ) method. A food intake frequency survey containing 103 food items was designed for the community-based cohort study, and its validity was evaluated with a revised version including three additional items was published after the second follow-up study [32,33]. The survey listed frequently consumed foods in Korea and asked participants to specify their average consumption frequency over the past year, using nine options ranging from “rarely” to “3 times a day” [34]. Using this data, the nutrient intake levels of 23 nutrients, including total energy intake, were calculated. In addition to our study, we constructed a dietary vitamin D database that allowed us to calculate dietary vitamin D (D2 + D3) levels using food items included in the SQFFQ and three nutrient databases. The methodology used for calculating dietary vitamin D intake is described in detail in a previous study [35]. To minimize the possibility of misclassification in the analysis, we calculated the mean frequency of dietary data from both the baseline and second follow-up surveys.

### 2.4. Phytochemical Index

Using the Food Composition Table 10.0 version of the National Institute of Agricultural Sciences under the Rural Development Administration and the Computer Aided Nutritional Analysis Program 5.0 of the Korean Nutrition Society [36,37], we constructed an updated nutrient database of phytochemical-rich foods, which included whole grains, fruits, vegetables, pulses, nuts, and seaweed [21,23,24,38]. To calculate the PI, the total calorie intake from dietary phytochemical-rich foods was divided by the overall daily calorie intake, as previously described in the literature [21,22]. Foods treated with salt or sugar, kimchi, powdered or seasoned nuts, or lacking proper information were excluded from the PI.

### 2.5. Bone Mineral Density Measurement and Definition of Osteoporosis

During baseline and follow-up examinations, study participants were interviewed by trained staff about their history of osteoporosis, treatment received, and medication use. Additionally, BMD was measured at baseline and during follow-up, three times each at the distal radius and midshaft tibia, using the Omnisense 7000s device (Sunlight Medical Ltd., Ramat Gan, Israel). The average of the speed of sound, T, and Z scores was calculated [28]. The incidence of osteoporosis was defined as a new diagnosis of osteoporosis by a physician, the initiation of treatment, or having a T-score of −2.5 or below at the follow-up examination [2,39].

### 2.6. Statistical Analysis

Categorical variables are presented as number and percentage and the significance of these variables was tested using the chi-squared test. Continuous variables are presented as mean ± standard error and significance was tested using general linear regression. The duration of follow-up was established as the time interval between the date of the baseline survey and the date of osteoporosis occurrence or the last known survival date for individuals without osteoporosis. To calculate the incidence rate, we divided the number of people who developed osteoporosis during the follow-up period by the sum of the years of follow-up. The association between PI and osteoporosis was analyzed using Cox proportional hazards regression and hazard ratios (HRs) with 95% confidence intervals (CIs). To determine the *p*-value for trend, a median value was calculated for each PI quantile and used as a continuous variable in the regression model. Potential confounders were determined through preliminary analysis and a literature review [40,41,42,43], but no effect modifiers were identified that affected the association between PI and osteoporosis in the fully adjusted model.

We constructed three models in our analysis: Model 1 (crude), Model 2 (age-adjusted), and Model 3 (adjusted for age, household income, education level, smoking status, alcohol consumption, physical activity, BMI, calcium, vitamin D, and total energy intake). To examine the dose-response relationship between PI and osteoporosis risk, we used restricted cubic spline regression with all covariates in Model 3. All statistical analysis was performed using SAS 9.4 (SAS Institute, Cary, NC, USA), and a significance level of α = 0.05 was set.

## 3. Results

### 3.1. General Characteristics of the Participants

During a 5.34-year mean follow-up, 707 and 1333 cases of osteoporosis were reported in premenopausal and postmenopausal women, respectively. The incidence rate of osteoporosis per 1000 person-years was 44.86 in premenopausal women and 151.66 in postmenopausal women. The participants were grouped based on the PI quartile for comparison of their characteristics in premenopausal and postmenopausal women (as shown in Table 1). The median PI for premenopausal women ranged from 7.45 (lowest quartile) to 23.33 (highest quartile), and from 8.00 (lowest quartile) to 22.04 (highest quartile) for postmenopausal women. In both premenopausal and postmenopausal women, a higher PI was associated with a higher education level (*p* = 0.03 for premenopause, *p* < 0.001 for premenopause), non-drinking (*p* < 0.001 for premenopause, *p* = 0.008 for postmenopause), and higher intake of calcium and vitamin D (all *p* < 0.001). Aging was significantly associated with higher PI in premenopausal women, while postmenopausal women with higher PI tended to have lower physical activity levels.

### 3.2. Association between PI and Osteoporosis Incidence

Table 2 shows the HR for incident osteoporosis according to PI in premenopausal and postmenopausal women. There was no significant association between PI and osteoporosis in premenopausal women (all *p*-values for trend > 0.05). However, in postmenopausal women, the association between PI and osteoporosis was significant in all three models. In the fully adjusted Model 3 analysis of postmenopausal women, the risk of osteoporosis was 16% lower in the highest PI quartile compared to the lowest quartile (HRs: 0.84, 95% CIs: 0.71–0.99), and the risk decreased with increasing PI quartile in a dose-response pattern (*p*-value for trend = 0.02).

### 3.3. Dose-Response Relationship between PI and Osteoporosis

**Figure 1:**
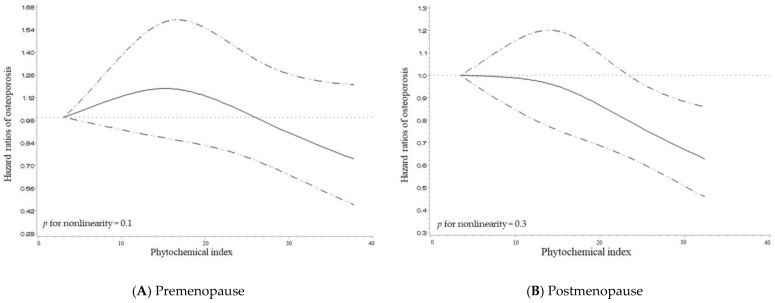
Multivariable adjusted hazard ratios (95% confidence intervals) for the non-linear relationship between the phytochemical index and osteoporosis stratified by (**A**) premenopause and (**B**) postmenopause. The model was adjusted for age, alcohol consumption, household income, education level, smoking status, physical activity, body mass index, and dietary intakes of total energy and calcium, and vitamin D.

Figure 1 shows the dose-response relationship between PI and osteoporosis as depicted in the spline curve. After adjusting for age, BMI, household income, education level, smoking status, alcohol consumption, physical activity, calcium intake, vitamin D intake, and total energy intake, the risk of osteoporosis appears to decrease as PI increases for both premenopausal and postmenopausal women. Although the PI appeared to have no association with osteoporosis in premenopausal women, the trend indicated a gradual decline in the risk of osteoporosis as the PI level reached 26. In contrast, for postmenopausal women, the risk of osteoporosis starts to decrease at a slightly lower point of PI level 10, with a more pronounced decrease compared to premenopausal women. The curve indicates that there is no evidence of non-linearity in the association between PI and osteoporosis for both premenopausal (*p*-value for nonlinearity = 0.1) and postmenopausal women (*p*-value for nonlinearity = 0.3).

## 4. Discussion

This study found that a high quantile of PI was associated with a reduced risk of developing osteoporosis in postmenopausal women. However, no significant relationship was observed between PI and osteoporosis risk in premenopausal women.

Osteoporosis can significantly impact the quality of life for older women [9,44]. The weakening and brittleness of bones can make even simple tasks such as standing or sitting upright painful and challenging [9]. The elevated risk of fractures can cause a loss of independence and mobility, leading to decreased self-esteem, confidence, and even depression or other mental health problems [45]. Physical limitations and increased pain due to osteoporosis can prevent older women from participating in enjoyable activities, causing feelings of isolation and loneliness [44,46]. Overall, osteoporosis has the potential to greatly lower the quality of life for older women, affecting both their physical and mental well-being. However, our findings indicate that incorporating a diet high in phytochemicals may decrease the risk of osteoporosis in older women, potentially enhancing their quality of life in the latter stages of their lives.

The levels of phytochemicals are largely influenced by fruits and vegetables, and there is conflicting evidence regarding their effect on bone health. The Women’s Cohort Study conducted in the United Kingdom, which involved 26,318 women aged between 35 and 69 years, showed results indicating that adhering to a vegetarian diet may pose a greater risk of hip fractures and osteoporosis when compared to individuals who consume meats and seafoods [10]. This highlights the potential harm a vegetarian diet may cause to bone health. However, other studies have found a positive association between vegetable and fruit intake and BMD in postmenopausal women. A meta-analysis of observational studies including 18 studies and 12,643 participants found that fruit consumption may have a protective effect on osteoporosis in postmenopausal women (odds ratio: 0.68; 95% CI: 0.56–0.83). Additionally, another meta-analysis of 10 studies and 14,247 participants revealed that a higher intake of vegetable-based diets was associated with lower odds of osteoporosis in postmenopausal women (odds ratio: 0.73; 95% CI: 0.57–0.95) [11,12]. These results suggest that increasing vegetable and fruit intake could potentially lower the risk of osteoporosis and highlight the importance of a balanced and nutrient-dense diet to maintain optimal bone health.

Prior literature has shown that various types of phytochemicals have a positive effect on BMD and osteogenesis and explained the underlying mechanisms [13,14,15,16]. For example, isoflavones have been shown to reduce the risk of osteoporosis by decreasing the expression of main protein factors that regulate bone metabolism, such as tumor necrosis factor-α and interleukin-6, which activate osteoclasts. In addition, isoflavones can also dose-dependently increase the expression of osteoprotegerin, which inhibits osteoclast formation [13,14]. Similarly, polyphenols have been found to decrease bone loss by exerting antioxidant and anti-inflammatory effects, improving osteoblastogenesis, and reducing osteoclastogenesis [15]. Polyphenols promote the differentiation and absorption of bone cells by eliminating free radicals and reducing cytokine damage, such as tumor necrosis factor-α and receptor activator of nuclear factor kappa-B ligand, which are inflammatory and osteoclast differentiation factors. Additionally, they can downregulate protein complexes such as nuclear factor kappa-light-chain-enhancer of activated B cells, which is involved in osteoclast differentiation [15]. These mechanisms suggest that polyphenols may help maintain bone health and prevent the development of osteoporosis. Furthermore, genistein, a specific type of polyphenol found in soybeans that also functions as a phytoestrogen, has been found to increase the number of cells in spine-derived bone cells by inhibiting protein tyrosine kinase, which is responsible for cell differentiation, proliferation, and death [16,17]. A randomized double-blind placebo-controlled study of postmenopausal women aged between 47 and 57 years found that genistein significantly increased the BMD in the femur and lumbar spine [47]. This finding provides evidence for the potential benefits of genistein and supports the idea that increasing the intake of specific types of polyphenols may promote bone health and help prevent the development of osteoporosis.

Studies have shown that lycopene can be beneficial for bone health, particularly in postmenopausal women. In a cross-sectional study of postmenopausal women aged 50–60 years, a significant positive association was found between dietary lycopene intake and blood lycopene levels, and women with high blood lycopene levels were found to have low protein oxidation and lower serum levels of markers of bone resorption [18]. Another study of postmenopausal women at St. Michael’s Hospital in Canada showed that restricting dietary lycopene resulted in reduced serum lycopene, lutein/zeaxanthin, α-/β-carotene, and antioxidant enzymes, and an increase in bone resorption markers, suggesting that lycopene plays a role in oxidative stress and bone resorption [19]. This provides evidence that lycopene’s antioxidant effects may influence BMD. Moreover, multiple epidemiological studies have identified an association between carotenoids and BMD. For example, a meta-analysis of 15 studies discovered an inverse association between β-carotene intake and osteoporosis in women and Asians, with odds ratios of 0.68 (95% Cl: 0.49–0.96) in women and 0.51 (95% Cl: 0.40–0.65) in Asia. In this meta-analysis, authors also reported a positive association between β-carotene intake and overall BMD (standard mean difference: −0.21, 95% Cl: −0.39–−0.03), suggesting that β-carotene may have a beneficial effect on bone health [48]. In addition to previous studies, a cross-sectional analysis of data from the NHANES 2005–2018 survey, which included 4820 people aged 50 or older, showed that individuals with higher β-carotene and β-cryptoxanthin intake had a lower prevalence of osteoporosis compared to those with lower intake. Furthermore, higher β-carotene intake was found to be associated with higher total hip and lumbar spine BMD [20]. These findings suggest that increasing the intake of foods that are rich in β-carotene and β-cryptoxanthin, such as carrots, sweet potatoes, and citrus fruits, may have a positive impact on bone health and potentially reduce the risk of osteoporosis in older adults. Our findings linking PI to osteoporosis in postmenopausal women are in line with prior studies and suggest that the multifaceted role of phytochemicals in plants may decrease the risk of osteoporosis and fractures [47,49,50].

The impact of dietary components on bone health is a multifaceted relationship subject to various factors such as the presence of oxalates, phytates, and insoluble fiber, all of which can limit calcium absorption [51,52,53]. For instance, oxalate, found in vegetables such as spinach, interacts with calcium to form calcium oxalate, a compound with low solubility, of which only a small fraction is absorbed by the body while the majority is excreted through feces [51]. Similarly, phytate, found in soybeans and related foods, can bind with calcium in the stomach, generating a water-soluble calcium-phytate complex that reduces calcium absorption [51,52]. Given the vast array of nutrients and chemical compounds present in fruits and vegetables, evaluating their cumulative effect on bone health may be a complex task. Consequently, future research should prioritize examining these interactions in order to gain a more comprehensive understanding of the role diet plays in maintaining bone health.

This study has some limitations to consider. First, there is a potential for underestimation of phytochemical intake levels due to the exclusion of certain food groups not captured by the SQFFQ. In addition, PI calculation is based on food source calories, leading to the possibility of underrepresentation or omission of low-calorie, phytochemical-rich foods. Second, there is a possibility of unmeasured or unknown confounding factors affecting the association, despite efforts to consider and adjust for multiple confounding factors identified through a review of previous literature and preliminary analysis. Third, since only the distal radius and midshaft tibia were assessed, the BMD in other regions such as the femoral neck and lumbar spine could not be evaluated. Finally, while our findings suggest that consuming phytochemical-rich foods may lower the risk of osteoporosis among post-menopausal women, the results might not be generalizable to individuals with different phytochemical intake levels, such as those following vegetable-based diets or vegetarians.

Despite its limitations, we used a holistic approach instead of examining a single nutrient, and to our knowledge, this is the first community-based prospective cohort study in Korea to investigate the association between phytochemical intake and osteoporosis. Furthermore, we used a validated SQFFQ that accurately reflects Korean eating patterns. Lastly, our inclusion of BMD measurements in addition to reporting may have reduced misclassification of our primary outcome variable.

## 5. Conclusions

In conclusion, this study provides evidence that incorporating phytochemical-rich foods into one’s diet has the potential to greatly reduce the risk of osteoporosis, especially in postmenopausal women. The findings of this study add to the growing body of literature supporting the role of dietary factors in promoting bone health. In particular, the study highlights the benefits of phytochemicals found in fruits and vegetables, which are an important component of a healthy diet. With an aging population, preventing osteoporosis is essential for improving the quality of life for those spending more time in their later years. This study lays the foundation for education on osteoporosis prevention, particularly for middle-aged and elderly women. Further research is necessary to fully understand the mechanisms behind the observed associations and to understand the role of diet in promoting bone health in the elderly. Nonetheless, this study lays the foundation for future research on osteoporosis prevention and provides a potential solution to improving the quality of life for older women.

## Figures and Tables

**Table 1:** Demographic and lifestyle characteristics of premenopausal and postmenopausal women according to quartiles of the phytochemical index.

	Quartiles of PI				*p*-Value
Characteristics	Q1	Q2	Q3	Q4	
Premenopausal					
No. of participants	688	689	689	689	
PI, median (range)	7.45 (0.27–10.27)	12.90 (10.28–15.24)	17.44 (15.25–19.70)	23.33 (19.71–64.22)	
Age (years)	47.65 ± 0.29	46.36 ± 0.25	47.48 ± 0.28	50.46 ± 0.34	<0.001
Household income					<0.001
Low or mid–low	346 (51.49)	283 (41.80)	250 (36.71)	332 (49.04)	
Mid–high or high	326 (48.51)	394 (58.20)	431 (63.29)	345 (50.96)	
Education level					0.03
High school graduation or lower	647 (94.45)	625 (91.11)	621 (90.39)	624 (91.50)	
College graduation or higher	38 (5.55)	61 (8.89)	66 (9.61)	58 (8.50)	
Alcohol consumption					<0.001
Non-drinkers	465 (67.78)	457 (66.62)	470 (68.61)	521 (75.95)	
Drinkers	221 (32.22)	229 (33.38)	215 (31.39)	165 (24.05)	
Smoking status					0.4
Non-smokers	629 (93.46)	644 (95.55)	653 (96.03)	651 (95.32)	
Former smokers	9 (1.34)	8 (1.19)	8 (1.18)	8 (1.17)	
Current Smokers	35 (5.20)	22 (3.26)	19 (2.79)	24 (3.51)	
Physical activity ¹					0.04
Low	257 (38.02)	209 (30.69)	242 (35.38)	211 (30.85)	
Mid	203 (30.03)	227 (33.33)	223 (32.60)	245 (35.82)	
High	216 (31.95)	245 (35.98)	219 (32.02)	228 (33.33)	
Body mass index (kg/m^2^)	24.75 ± 0.12	24.82 ± 0.12	24.57 ± 0.12	24.75 ± 0.13	0.7
Calcium intake (mg/day)	382.56 ± 7.36	478.82 ± 8.64	503.36 ± 9.26	529.41 ± 9.63	<0.001
Total energy intake (kcal/day)	1723.23 ± 20.15	1930.91 ± 22.43	1899.72 ± 21.51	1832.15 ± 21.64	0.003
Vitamin D intake (μg/day)	1.63 ± 0.05	2.18 ± 0.05	2.22 ± 0.06	2.23 ± 0.07	<0.001
Postmenopausal					
No. of participants	461	461	462	461	
PI, median (range)	8.00 (0.67–10.63)	12.99 (10.63–15.03)	17.02 (15.05–19.15)	22.04 (19.17–46.42)	
Age (years)	58.38 ± 0.32	57.62 ± 0.32	58.22 ± 0.32	58.32 ± 0.32	0.8
Household income					0.01
Low or mid–low	351 (77.65)	342 (75.50)	310 (69.35)	320 (70.64)	
Mid–high or high	101 (22.35)	111 (24.50)	137 (30.65)	133 (29.36)	
Education level					<0.001
High school graduation or lower	448 (99.12)	446 (97.38)	429 (93.46)	439 (95.85)	
College graduation or higher	4 (0.88)	12 (2.62)	30 (6.54)	19 (4.15)	
Alcohol consumption					0.008
Non-drinkers	342 (74.67)	365 (79.52)	368 (80.17)	382 (83.77)	
Drinkers	116 (25.33)	94 (20.48)	91 (19.83)	74 (16.23)	
Smoking status					0.08
Non-smokers	429 (93.87)	437 (95.62)	430 (94.51)	430 (94.30)	
Former smokers	3 (0.66)	5 (1.09)	12 (2.64)	7 (1.54)	
Current Smokers	25 (5.47)	15 (3.28)	13 (2.86)	19 (4.17)	
Physical activity ¹					<0.001
Low	138 (30.20)	138 (30.13)	162 (35.37)	165 (35.95)	
Mid	130 (28.45)	161 (35.15)	152 (33.19)	173 (37.69)	
High	189 (41.36)	159 (34.72)	144 (31.44)	121 (26.36)	
Body mass index (kg/m^2^)	24.87 ± 0.15	25.25 ± 0.15	25.32 ± 0.16	24.94 ± 0.16	0.7
Calcium intake (mg/day)	336.16 ± 8.11	428.50 ± 9.00	465.40 ± 10.30	506.26 ± 11.71	<0.001
Total energy intake (kcal/day)	1611.82 ± 21.13	1790.18 ± 21.81	1780.44 ± 21.90	1751.72 ± 23.90	<0.001
Vitamin D intake (μg/day)	1.15 ± 0.05	1.58 ± 0.06	1.71 ± 0.06	1.78 ± 0.07	<0.001

PI, phytochemical index; Q, quartile. Values are mean ± standard error or n (%). *p*-values were derived from a x² test for categorical variables and from generalized linear regression analysis for continuous variables. ¹ The level of physical activity was calculated by metabolic equivalent tasks per week (METs-h/week) and categorized into tertiles.

**Table 2:** Hazard ratios (95% confidence intervals) for osteoporosis according to the dietary phytochemical index quartile in premenopausal and postmenopausal women (*n* = 4600).

	Quartiles of PI				
	Q1	Q2	Q3	Q4	*p* for Trend
Premenopause					
No. of participants	688	689	689	689	
No. of cases (%)	164 (23.84)	193 (28.01)	182 (26.42)	168 (24.38)	
Model 1	Ref	0.99 (0.81–1.22)	0.99 (0.80–1.22)	1.12 (0.90–1.39)	0.3
Model 2	Ref	1.12 (0.91–1.38)	1.00 (0.81–1.23)	0.84 (0.68–1.05)	0.9
Model 3	Ref	1.15 (0.92–1.44)	1.16 (0.93–1.46)	0.98 (0.78–1.24)	0.8
Postmenopause					
No. of participants	461	461	462	461	
No. of cases (%)	348 (75.49)	335 (72.67)	329 (71.21)	321 (69.63)	
Model 1	Ref	0.95 (0.82–1.10)	0.89 (0.76–1.03)	0.85 (0.73–0.99)	0.03
Model 2	Ref	1.02 (0.87–1.18)	0.90 (0.77–1.05)	0.85 (0.73–0.99)	0.01
Model 3	Ref	1.02 (0.87–1.19)	0.92 (0.78–1.08)	0.84 (0.71–0.99)	0.02

Q, quartile; Ref, reference. Model 1, unadjusted; Model 2, adjusted for age (continuous); Model 3, adjusted for age (continuous), household income (low or mid–low and mid–high or high), education level (high school graduation or lower and college graduation or higher), smoking status (non-smokers, former smokers, and current smokers), alcohol consumption (non-drinking and drinking), physical activity (low, mid, and high), body mass index (continuous), calcium intake (continuous), vitamin D intake (continuous), and total energy intake (continuous).

## Data Availability

Restrictions apply to the availability of these data. Data was obtained from the Korean Genome and Epidemiology Study (KoGES) and are available from the National Institute of Health (NIH) at http://nih.go.kr/NIH_NEW with the permission of KoGES.
